# Contributions to the dynamics of cervix remodeling prior to term and preterm birth^†^

**DOI:** 10.1095/biolreprod.116.142844

**Published:** 2016-12-22

**Authors:** Steven M. Yellon

**Affiliations:** Longo Center for Perinatal Biology, Departments of Basic Sciences Division of Physiology and Pediatrics, Loma Linda University School of Medicine, Loma Linda, CA 92350, USA

**Keywords:** cervix, parturition, progesterone/progesterone receptor, macrophage, extracellular matrix

## Abstract

Major clinical challenges for obstetricians and neonatologists result from early cervix remodeling and preterm birth. Complications related to cervix remodeling or delivery account for significant morbidity in newborns and peripartum mothers. Understanding morphology and structure of the cervix in pregnant women is limited mostly to the period soon before and after birth. However, evidence in rodent models supports a working hypothesis that a convergence of factors promotes a physiological inflammatory process that degrades the extracellular collagen matrix and enhances biomechanical distensibility of the cervix well before the uterus develops the contractile capabilities for labor. Contributing factors to this remodeling process include innervation, mechanical stretch, hypoxia, and proinflammatory mediators. Importantly, the softening and shift to ripening occurs while progesterone is near peak concentrations in circulation across species. Since progesterone is required to maintain pregnancy, the premise of this review is that loss of responsiveness to progesterone constitutes a common final mechanism for remodeling the mammalian cervix in preparation for birth at term. Various inputs are suggested to promote signaling between stromal cells and resident macrophages to drive proinflammatory processes that advance the soft cervix into ripening. With infection, pathophysiological processes may prematurely drive components of this remodeling mechanism and lead to preterm birth. Identification of critical molecules and pathways from studies in various rodent models hold promise for novel endpoints to assess risk and provide innovative approaches to treat preterm birth or promote the progress of ripening at term.

## Summary Sentence

The mechanism for cervix remodeling in preparation for birth is proposed to involve a convergence of inputs and complex orchestration of inflammatory processes that direct resident macrophage activities to drive extracellular matrix degradation.

## Preterm birth is a significant clinical problem—Why study the cervix?

According to the World Health Organization and US Center for Health Statistics, the annual rate of preterm birth exceeds or is near 10% of all pregnancies in the majority of countries [1,2]. Premature birth, i.e., before 37 weeks of gestation, is the leading cause of mortality in the first year of life and is associated with increased risks for morbidity, as well as life-long cognitive deficiencies [3]. Common motifs leading to preterm births have not emerged from known risk factors such as genetics, infection, multiple pregnancy, a short cervix, cervical insufficiency, and idiopathic causes [4]. What is known is that preterm birth cannot occur without remodeling of the cervix. More than a decade ago, Iams et al. stated that “Theories of premature labor based on an understanding of the cervix as uniformly competent may underestimate the importance of the cervix, and overestimate the role of uterine activity, in the pathogenesis of prematurity” [5].

Quintessentially, the cervix serves as gatekeeper for the birth process given its functions as a barrier to protect development of the fetus within the uterus from the vaginal biome ecology [6]. This barrier also presents an impediment for birth. The cervix then acts as a gate with little resemblance to the collagen-dense structure that predominates for most of pregnancy and in nonpregnant women. In pregnant women, a “short” cervix of 25 mm or less at 24 or 28 weeks gestation is a sentinel for increased risk of preterm birth [7]. Progesterone supplementation and cerclage, within guidelines, have proven useful to manage risk for preterm birth in women diagnosed with a short cervix [7,8]. While such treatments in response to predisposing risks or symptoms are the best available approach to prolong pregnancy, the incidence of preterm birth remains relatively unchanged outside a limited subpopulation of patients [7,9]. Lack of cervix biopsy material from women prior to preterm or term labor limits understanding of the etiology of a short compared to a typical lengthened cervix of about 40 mm at term. Evaluation of cervix length by ultrasound or Raman spectroscopy, as well as analyses of cervix secretions, hold promise for risk assessment [4,10–12]. Yet, understanding molecules and cells that regulate aspects of remodeling are needed to diagnose and forestall preterm birth as premature labor is often the first symptom of a clinical problem [13–15].

Accordingly, this article reviews insights derived from experimental rodent models to provide perspective for the development of novel therapeutic strategies to track the progress of cervix remodeling and their potential usefulness to prevent preterm birth. The emerging consensus across species suggests that physiological inflammation is part of a final common mechanism that ripens the cervix independent of a fall in systemic progesterone concentrations and well before the labor at term or in preterm birth.

## Defining structural, biochemical, and mechanical characteristics of cervix remodeling

Although biopsy material from pregnant women before term is limited, longitudinal sections of the entire cervix in rodents have facilitated a comparative survey of the morphological heterogeneity through the external os and transition zone, into the lower uterus [16–19]. As outlined in a cogent review by the Word group in 2007, there are three phases of prepartum cervix remodeling [20]. Phase 1 is characterized by softening of the cervix with a progressive increased turnover of the extracellular matrix, a decline in cross-linked collagen, and a relative decrease in collagen concentration due to water imbibition. Phase 2 is defined as ripening because collagen structure further degrades as the cervix loses gross morphological features; uterine features like glands and smooth muscle are not present. In phase 3, the cervix dilates, morphological features disappear to efface with the uterus, and ultimately labor results in birth. Although there are some studies in rodents during softening and ripening, no report focuses on human cervix morphology during the shift from phase 1 to 2 of remodeling.

The cervix is a dynamic structure with respect to the area of luminal epithelium, blood vessels, stroma, and the extracellular collagen matrix [21]. Comparisons of cervix biopsies from women in the late third versus first trimester indicate a decline in extracellular collagen and structure [22,23], although no phase of remodeling is thought to be driven solely by increased collagenase activity [24]. Extractable soluble collagen near term does not incorporate into fibrillary cross-linked structure [25]. In the cervix of pregnant rats, reduced hydroxyproline concentrations parallel increased distensibility, and extracellular collagen structural disorganization with progression to term as determined by light-induced fluorescence [26]. To address the possibility that degradation of collagen structure occurs before ripening, other approaches are needed to study the transition from a soft to ripening cervix.

Biochemical studies of collagen in the cervix require cell dispersal. Anatomical integrity is lost with dispersion and mixing of cells from a heterogeneous morphology of vascular, stroma, some smooth muscle, and epithelium that change as pregnancy progresses to term. Among various dyes that have been used to stain collagen in fixed tissues, picrosirius red (PSR) stain is specific for collagen types I and III across many species and tissues including liver, kidney, and lung [27–30]. Moreover, PSR stains collagen fibers in the extracellular matrix of cervix in women and rodents [31–33].The assessment of optical density of birefringent circular polarized light in PSR-stained cervix sections has proven useful to identify loss of structure been in several murine strains [34–37], two rat strains [17,38], and more recently in a study of cervix biopsies from women at term and preterm birth [23]. The latter study also confirmed that hydroxyproline concentration and extracellular PSR-stained collagen were reduced in cervix biopsies from women at term compared to tissue collected from women prior to term.

Cross-linked collagen is characterized by image analysis of PSR stain in sections from pathological tissues [39,40]. Electron microscopic studies in the cervix of rats collectively indicate that interfibrillary distance between collagen fibers increases while fibril diameter and length decline following prostaglandin or progesterone receptor (PR) antagonist treatment, as well as with the progress of pregnancy [16,18,41]. Findings in these studies further suggest that decreased concentration of organized collagen, i.e., decreased hydroxyproline concentrations and increased soluble collagen that is not cross-linked, correlates with disorientation of fibril. These data clearly indicate a degradation of collagen structure in the extracellular matrix of the prepartum cervix during the transition from soft to ripening (from phase 1 to 2). In addition, fewer, shorter, and thinner fibers provide a compelling rationale for a degradative process in which cross-linked collagen content declines or fails to be restored as the cervix ripens before birth both preterm and at term.

The structural organization of collagen in the extracellular matrix described above directly corresponds with increased compliance in mechanical properties of the cervix in rodents [24,42–44]. Phase 1 softening includes decreased stiffness and increased extensibility on day 16 versus day 10 postbreeding [24]. Phase 2 follows phase 1 during the period of ripening from day 16 to prepartum day 22 in rats, which is characterized by similar resilience to stretch and distensibility, but greater dispersal of collagen into fine, poorly oriented fibers in the extracellular matrix [16,41]. Thus, the switch from phase 1 softening to phase 2 ripening is a critical period for cervix function.

## Systemic progesterone loss does not drive cervix remodeling

**Figure 1.**
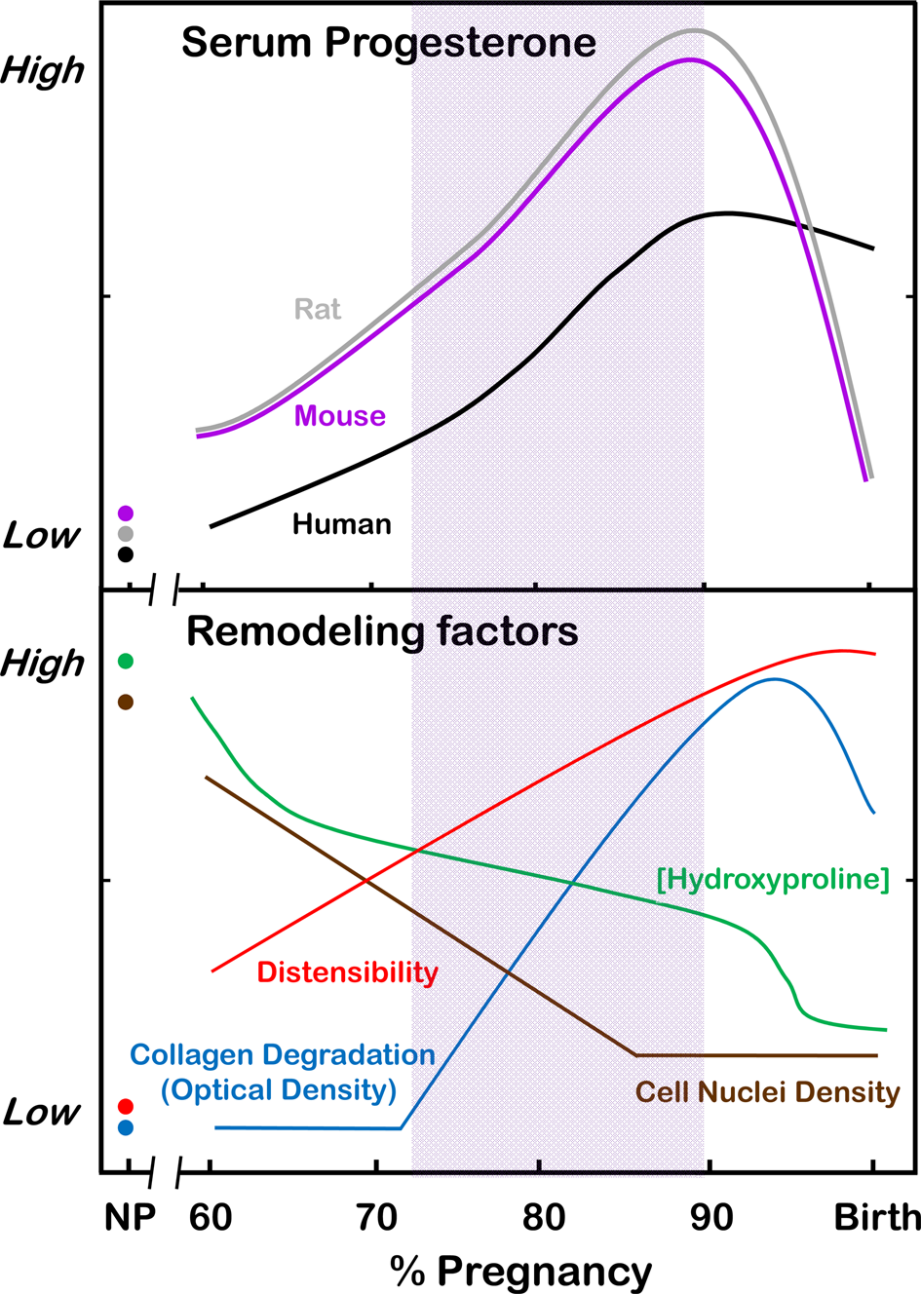
**Top**. Systemic progesterone as % of pregnancy relative to term of approximately 23 days for rats, 19 days for mice, and 40 weeks for humans [42,46,139]. **Bottom**. Remodeling characteristics of the rodent cervix as % of term pregnancy as indicated by morphology (cell nuclei density indication of cellular hypertrophy and edema), extracellular collagen degradation (structure and cross-linked content: hydroxyproline and optical density of PSR stained collagen birefringence) [34–36,140], and compliance (distensibility). Compliance is a biomechanical term that reflects the decreased slope of cervix stretch associated with disarrayed collagen fibers, elastic extension, increased water content, and increased proteoglycans [18,24]. Peak rate of change in cervix remodeling (shaded area) reflects shift from phase 1 soft to phase 2 ripening cervix [24] between 75% and 90% of pregnancy. During this transition, serum progesterone concentrations are 5- to 8-fold higher than the estrus cycle peak in nonpregnant individuals (NP). Comparable data for cervix remodeling in humans are not available before 95% of term pregnancy.

Progesterone is essential for maintaining pregnancy. Csapo proposed in 1956 that pregnancy terminates from withdrawal of a “progesterone block” [45]. This conclusion was based, in part, on evidence that systemic progesterone falls prior to the onset of labor in many species including most rodents, rabbits, sheep, and some primates, but not in women, apes, and guinea pigs (Figure 1, top) [46,47]. A common misconception comes from extending this species difference in serum progesterone at term before labor to the earlier time period, during phases 1 and 2, because neither systemic nor cervix content of progesterone declines when the cervix transitions from soft to ripening (Figure 1, bottom). Specifically in mice, systemic and cervix progesterone remain near peak by day 17 postbreeding, 2 days before birth [42,46]. Moreover, serum progesterone concentrations on the day before birth in rodents actually exceeds reported requirements to saturate tissue PRs [48]. Thus, evidence that systemic progesterone is sustained during the remodeling process well before term across species focuses attention on insights about commonalities in the mechanism for prepartum remodeling of the cervix.

## Inflammation and the drive of term and preterm cervix remodeling: insights for humans from rodent models

In general, the morphology that defines cervix remodeling at term or with preterm birth resembles an inflammatory process [49–52]. An increased presence of immune cells in the lower uterine segment of parturient women [31,52,53] was recently confirmed in a study of peripartum cervix biopsies, which found a greater density of macrophages in the cervix from women during preterm birth, as well as at term, whether or not in labor [23]. The similar increase in resident macrophages along with reduced cell nuclei density in the cervix of women who gave birth whether preterm or at term suggests premature advance in the mechanism that regulates these characteristics of remodeling. Although cervix biopsy tissue of earlier phases of remodeling is unavailable in women, characteristics of cervix morphology have been studied in a mouse model for pregnancy and in mice and rats during phases of remodeling [54]. Nonpregnant mice given progesterone and estradiol to simulate the endocrine environment during pregnancy had reduced cell nuclei density, decreased extracellular collagen, and enhanced presence of macrophages in the cervix on 15–18 days posttreatment. These morphological characteristics are consistent with the timeline associated with the transition from a soft to ripe cervix in pregnant mice 2.5–4 days before birth [35,55–57].

**Figure 2.**
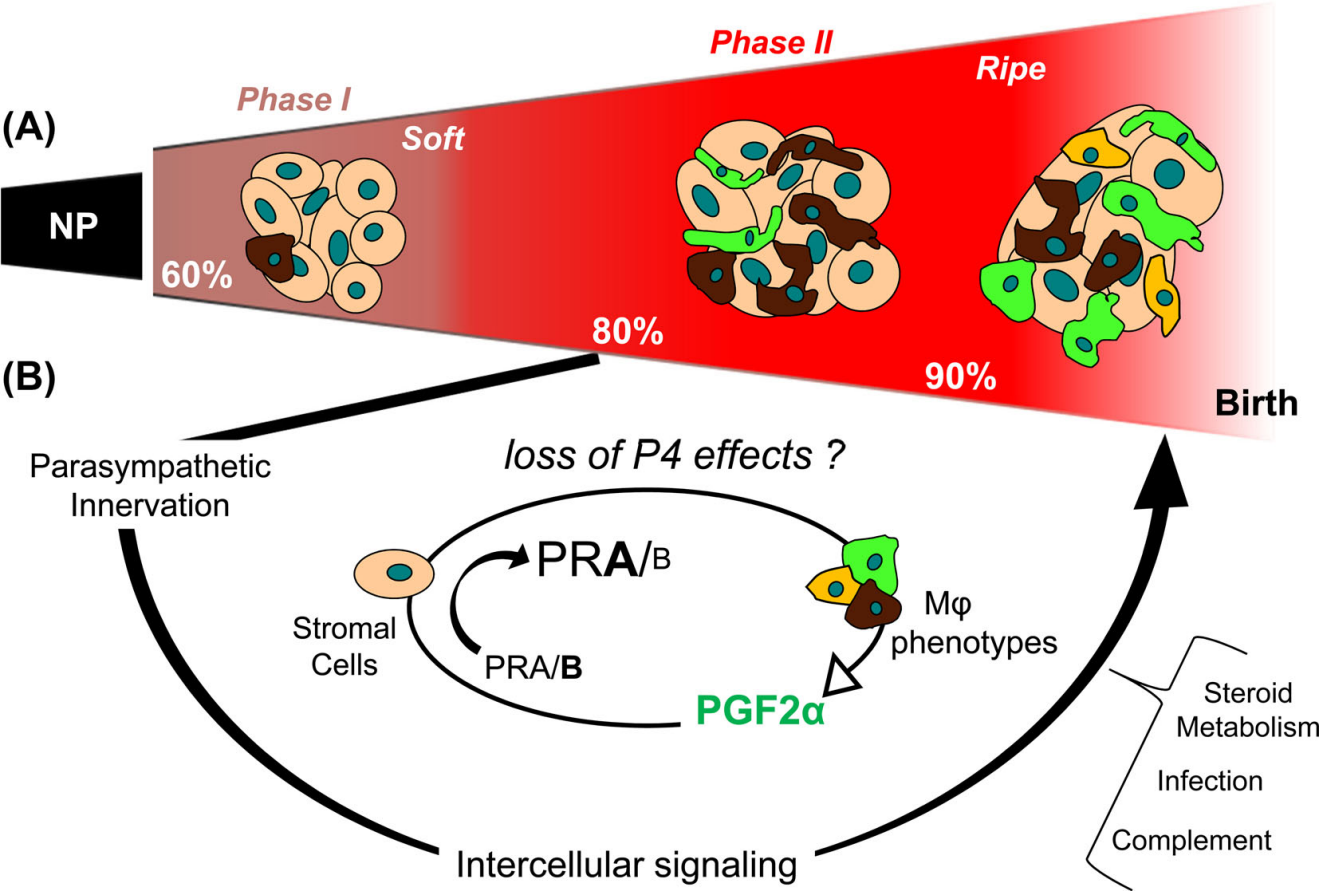
Schema of a final common pathway for cervix remodeling that involves the convergence of various contributing factors to regulate local functional withdrawal of progesterone actions. (**A**) Based on evidence in rodent models with insights from findings in prepartum women [20], the cervix during phase 1 of remodeling with progestational support grows and softens from a nonpregnant (NP) tightly closed dense collagen cross-linked structure. Density of cell nuclei is high while macrophages in stroma are relatively low when the cervix is maximally soft. Subsequently, phase 2 ripening reflects reduced cell nuclei density and increased residency by macrophages with divergent phenotypes that are proposed to facilitate degradation of the extracellular matrix, sustain tensile strength [36], and promote distensibility [42,75,141]. (**B**) Multiple factors that include neural and proinflammatory stimuli are proposed to regulate the timing and pace of remodeling. Critical for phase 2 ripening is the loss of progesterone effects to sustain a soft cervix. Cells that contain genomic PRs [35,142] in the stroma, in proximity to most resident to macrophages, and possibly luminal epithelia would integrate local signals to guide phenotypic activities and prostaglandin metabolism [143]. Macrophages are a possible source for prostaglandin production. Increased prostaglandin F2α (PGF2α) production has been found to upregulate PR-A [111], a possible mechanism to induce a functional progesterone withdrawal in the cervix between about 75% and 85% of pregnancy. Conceivably, the crosstalk between stroma cells and macrophages could be mediated by paracrine signals that include proinflammatory and phagocyte-related cytokines, chemokines, hypoxia-linked molecules, as well as nitric oxide, prostaglandins, and VEGF to advance extracellular collagen matrix degradation before the uterus develops contractile capabilities for labor. Progesterone withdrawal, as possibly defined by a shift in PR-A/B isoforms in stromal cells and the actions of prostaglandins, is proposed to drive the ripening process by guiding local macrophage actions. The role of complement, local steroid metabolism, and mechanical stretch may be part of the final common pathway for the physiology of remodeling at term. Along with proinflammatory stimuli (infection) and other pathophysiological input, a threshold may be exceeded to accelerate preterm cervix remodeling and preterm birth.

Across viviparous species, the transition from a soft to ripe cervix is characterized by increased vascular permeability and elevated concentrations of proinflammatory mediators [58], as well as degradation of extracellular cross-linked collagen [17,53] (Figure 2). These remodeling changes are represented in the schema that depicts the transition from a dense cross-linked collagen extracellular matrix to a less structured and distensible ripened structure before dilation (Figure 2A). Replicable evidence indicates an increased presence of macrophages in the cervix stroma in mice and rats 2–5 days before the day of birth compared to earlier in pregnancy or in nonpregnant controls [17,55,59]. These reports compared cellular and structural morphology of cervix in phases 1 and 2 of remodeling, e.g., softening on day 15 versus ripening by day 18 postbreeding. A study of the intervening period recently found that cell nuclei density declined and macrophage presence increased in the cervix by 16.5–17 days postbreeding–2 days or more preceding birth at term [54]. These studies suggest that increased macrophage presence in the cervix precedes the maximal increase in compliance that does not occur until the time of birth.

The presence of macrophages in tissue does not, by itself, explain its potential role in cervix function. Macrophages have a heterogeneity of activities that reflect local signals and specialized functions in different anatomical locations [60,61]. Insights about macrophage activation have advanced beyond the M1 (classical inflammatory)/M2 (alternative activated) classification [62–64]. Hume proposed that distinct populations of macrophages have an expression signature for a functional repertoire of activities that depend on local signals in each tissue [65]. With evidence that macrophages help to restructure the extracellular matrix in other tissues by phagocytosis and angiogenesis [66], the implication is that phase 2 ripening may result from the increased presence of macrophages [67,68]. To understand the importance of macrophage phenotypes during remodeling, a flow cytometry study of dispersed cervix after systemic perfusion was performed. Findings confirm immunohistochemical studies and extend insights to indicate that other macrophages with phenotypes indicative of inflammatory and phagocyte-related activities were increased in the prepartum cervix on the day before birth (day 18 postbreeding) compared to 3 days earlier on day 15 [69].

Although methodological differences between studies may account for the prepartum increase in macrophages in the cervix in one study, but not others, accuracy of resident macrophages improved when mice were perfused and a gating strategy for flow studies was standardized. Perfusion of mice was found to be necessary to exclude systemic immune cells and to focus on resident macrophages in the cervix compared to other reports [70,71]. Evidence that Ly6C^+^ cells (a systemic monocyte precursor for macrophages) are increased in cervix on day 18.75 compared to day 15 postbreeding suggests that more blood was present in prepartum samples, a consequence of increased size and vascularity of the ripened cervix [17,38]. In other tissues, monocytes that infiltrate into tissue typically differentiate into resident macrophages [72,73]. A cell viability marker rather than morphological forward- and side-scatter criteria for setting gates is needed to exclude nonspecific large debris or nonviable cells [74]. Accuracy of macrophage counts in the cervix was facilitated by comparisons with gate settings in runs of dispersed spleen, an organ rich in differentiated macrophages. In addition, some studies based conclusions on observations without actually counting macrophages in the sections of cervix [75,76]. This approach does not take into consideration the diverse distribution of macrophages across a heterogeneous morphology. Overall, the trajectory of available evidence suggests that there is an increase in resident macrophages with a spectrum of activities that involve a unique combination of proinflammatory, anti-inflammatory, and extracellular matrix repair activities and molecules [77].

Macrophages are not the only leukocyte in the cervix during pregnancy. However, the importance of the small populations of mast cells and other granulocytes that reside in the cervix for remodeling has yet to be realized [78,79]. Neutrophils are more abundant in the cervix of women near term and in labor [80], as well as in mice within 8 h of birth whether untreated at term or in models of preterm birth [35,81]. The increased peripartum presence of neutrophils [19,37], along with eosinophils [70,82], in the stroma comes well after the transition to ripening (late in phase 2) and may reflect some role relative to active labour or postpartum restoration to an unremodeled state in preparation for postpartum reproductive cycles.

Other contributing factors promote the transition from a soft to ripe cervix. Multiparous pregnancies are associated with increased risk of preterm birth [83]. However, whether growth of the womb affects biomechanical or structural characteristics of the cervix is not known. Increased pressure on the cervix as the singleton fetus develops or with multiple pregnancies could reduce blood supply and lead to hypoxic drive of proinflammatory processes that involve molecules like hypoxia-inducible factor-1α (HIF-1α) [84]. Since reduced oxygen activates macrophages [85], hypoxia along with chemokine and cytokine signals might direct local macrophage activities toward ripening.

## Contribution of rodent models to understanding preterm cervix remodeling in preterm birth

Investigations in rats have advanced understanding of the importance of innervation for cervix remodeling. The cervix in women [86] and rodents [87,88] is not only well innervated, but nerve fiber density is sustained or increased at term. Transection of the parasympathetic nerves that project to the cervix (at either the esophageal segment of the extraspinal cord vagus nerve or the pelvic nerve or both) delays birth and interfered with the typically increased presence of macrophages at term [38]. These parasympathetic pathways contain sensory neuropeptidergic fibers that are recognized to promote local vasodilation and inflammation [89]. In the cervix, neuropeptidergic fibers are present with increased density by the day before birth [37,90]. Moreover, central connections from the paraventricular nucleus to the cervix may participate in the circadian system control of the time of day of birth in mammals [88,91]. These findings raise the possibility that neural regulation of local inflammatory processes contribute to ripening.

Other rodent models have advanced understanding of factors that contribute to remodeling and parturition. Although genomic alterations in mouse models can be associated with fetal demise during pregnancy, mice that lack genes for molecules that are prominent in the remodeling or parturition processes are fertile and give birth at term [92,93]. Viable litters are produced by females lacking oxytocin, inducible nitric oxide synthase, corticotropin-releasing hormone, or the classic glucocorticoid receptor. In addition, mice lacking decorin, a proteoglycan implicated in extracellular matrix remodeling and capable of stimulating proinflammatory modulators by macrophages [94], are fertile and more than 90% deliver viable pups at term [95]. In addition, the hormone relaxin is critically important for softening of the cervix and contributes to extracellular matrix remodeling of the cervix [96,97]. However, the effects on the parturition process are less clear since most relaxin null mice deliver viable pups at term. Each of these molecules may be important contributing factors to the remodeling process or part of a convergent upstream input.

Another genetically altered strain progresses through pregnancy, but fails to deliver. Mice lacking anthrax toxin receptor 2 (Antxr2) do not give birth even though serum progesterone declines at term as in controls [44,98]. Insufficient ripening in *Antxr2*^−/−^ mice appears to result from dense deposits of proteins and disrupted collagen structure from reduced metalloproteinase and extracellular matrix turnover. Though immune cells in the cervix were not studied, there is evidence that Antxr2 regulates phagocytic and signaling activity by macrophages [99,100]. This finding raises the possibility that macrophage-related actions may be essential for extracellular matrix degradation and remodeling.

Another mouse model emphasizes the importance of steroid metabolism and a loss in progesterone action for parturition because removal of the ovaries overrides the parturition defect and induces birth within 24 h. In mice lacking 5 alpha-reductase type 1 (5αR1), more than 70% fail to deliver at term [42]. A well-reasoned rationale indicates that sustained actions by progesterone contribute to this parturition defect. However, similar distensibility properties, lack of histological differences, and comparable progesterone concentrations in both serum and cervix in *5αR1^−^^/^^−^* and wild-type mice during the transition from soft to ripening before day 17 postbreeding (phases 1 and 2, 75%–90% of pregnancy) do not implicate an effect of 5αR1 reductase on remodeling of the cervix at term. This does not exclude the possibility that *5αR1* may have a role in other aspects of ripening. A role for local metabolism of progesterone in inflammation-induced preterm birth is suggested by the effects of the proinflammatory cytokine IL-1B to increase expression of 20-α hydroxysteroid dehydrogenase in human cervical fibroblasts [101]. Further investigation is needed to understand the role of steroid catabolism for remodeling as *5αR1* is localized to cervix epithelium in mice, but found only in the stroma of women [102]. In this study, other enzymes involved in steroid metabolism in cervix biopsies from prepartum term compared to nonpregnant women suggest that a complex relationship between cervical epithelium and stroma may regulate local concentrations of progesterone and estrogen.

An essential contributor to parturition is recognized in mice that lack the prostaglandin F2α receptor (Ptgfr) where successful gestation is not followed by birth [34]. Compared to wild-type controls, characteristics of cervix remodeling including reduced extracellular collagen, increased presence of macrophages, and enhanced density of nerve fibers are similar in *Ptgfr^−^^/^^–^* mice as term approaches. The failure of *Ptgfr^−^^/^^–^* mice to delivery may result from sustained ovarian progesterone production due to failed luteolysis [103] and loss of systemic progesterone leads to birth within 24 h. These findings do not necessarily exclude a role for prostaglandins in the remodeling processes because prostaglandins are used for induction of labor in women [104,105]. Moreover, vaginal prostaglandin treatment of women near term increased cervix proteoglycan metabolism, decreased length, and produced a higher Bishop score (ripening index) [106,107].

Moreover in mice, delivery at term does not occur when enzymes required for production of prostaglandins are absent, i.e., Cox-1 or PLA2 [108,109]. Short-term effects of prostaglandin treatments on serum progesterone or cervix remodeling characteristics have yet to be studied, but evidence in rodents indicates that biomechanical changes after PGE2 treatment are similar to those during physiologic ripening [24]. The conclusion that prostaglandins induce preterm cervix remodeling and birth through a different mechanism than at term [81] awaits replication using groups that compare the same time points following treatment during the critical phases for remodeling. A crucial insight from these studies is that findings do not exclude the possibility that prostaglandins have a direct effect on cervix morphology. A time course study of the effects of prostaglandin agonist/antagonist treatment on serum progesterone and cervix morphology remains to be done.

Finally, the importance of the PR-A isoform for sustaining pregnancy and for cervix remodeling comes from the study of mice that lack the classic PR-B isoform [36]. In contrast to PR-A null mice that are infertile due to a defect in ovulation, homozygote matings of PR-B null mice are fertile [110]. With only PR-A present, the decline in cell nuclei density, the degradation of extracellular collagen, and the prepartum increase in macrophages in the cervix stroma were the same as in wild-type controls with the approach of term. Thus, in the absence of the PR-B isoforms, PR-A was capable of mediating all trophic actions of progesterone to support pregnancy and softening during phase 1 of remodeling. The premise that PR-A is involved in the transition to phase 2 ripening draws indirect support from the report by Mesiano and colleagues that prostaglandin 2α stimulates PR-A expression by human myometrial cells [111]. The potential mechanism for PR-A to block progestational actions of progesterone comes from the effects of PR-A as a negative repressor of PR-B [112]. Although PR isoforms have yet to be studied in the cervix of rodents, transcriptome analyses of the guinea pig uterine cervix indicate a downregulation of PR message late in pregnancy compared to mid-pregnancy [113]. Thus, a reasonable extension of this possibility is that an increase in the PR-A/PR-B ratio may block local trophic effects of progesterone to drive proinflammatory activities for ripening the cervix at term and possibly in inflammation-induced preterm birth.

## Functional loss of progesterone leads to cervix ripening and parturition at term and preterm birth

The possibility that a local functional progesterone withdrawal is part of the final common mechanism for cervix remodeling and parturition (Figure 2B) extends from Csapo's hypothesis about labor and parturition [45]. The premise is that withdrawal of progesterone's effect to sustain pregnancy and a soft cervix results in ripening and parturition across species. Collective circumstantial evidence has accumulated in support of these hypotheses: (1) loss of systemic progesterone production by ovariectomy induces remodeling changes in the cervix well in advance of labor [54]; (2) PR antagonist treatment induces remodeling and preterm birth in women and rodents [17,54,114]; (3) in rodents, biomechanical properties of the rodent cervix during ripening at term, i.e., increased extensibility, compliance, and strength, are similar to those induced by PR antagonist treatment [18,24,75,81]; (4) in women, PR antagonists also increase proinflammatory mediators, degrade extracellular collagen structure, enhance presence of immune cells in the prepartum cervix [115,116]; and (5) through genomic actions, the pure PR agonist R5020 inhibits ripening and delays preterm birth in pregnant rats when treated vaginally [117], and following ovariectomy in mice [54]. This is directly relevant for women, where progestogen treatments reduce the risk of preterm birth in some circumstances, a finding that may depend on systemic versus vaginal administration and study demographics [8,118–121]. Although the effects of these PR agonist treatments on cervix morphology in women are not known, the efficacy of genomic PR modulators and lack of change in the abundance of PR-stained cells in the cervix during peak remodeling in rodents [33,122] raise the possibility that PR-A or an inhibitor of PR function may be essential to the remodeling process. Such PR-A-mediated actions are unlikely to directly act on macrophages in the cervix, which lack PRs, but on stromal cells that abundantly stain for PR [17,122].

Focus on the interactions between stromal cells and macrophages during the transition from soft to ripening, phase 1 to 2, will advance our current understanding of prepartum cervix remodeling. Integration of various contributing factors by stromal cells, which in turn guide local phenotypic differentiation of macrophages, could orchestrate the degradation of extracellular collagen for ripening (Figure 2A), as well as coordinate postpartum regeneration of the cervix. The intimate communication among epithelial, stromal, immune, and vascular cells is likely to involve proinflammatory cytokines, chemokines, neuromodulators, HIF-1α, and prostaglandins that are recognized in other tissues to promote macrophage activities that lead to distensibility of the cervix for ripening. By example, in skin, progesterone promotes macrophage infiltration, wound healing activities, phagocyte-associated actions, and production of cytokines that regulate the extracellular matrix [123].

For preterm birth, the effects of inflammation are widely studied in the pregnant mouse models as proxy for the clinical scenario of intrauterine infection in pregnant women. A common model for inflammation-induced preterm birth uses the bacterial endotoxin lipopolysaccharide (LPS) that acts through Toll-like receptor-4 in cells in the cervix, including macrophages [124–129]. LPS treatment advances cervix remodeling and initiate labor within 24 h of treatment [81,130,131]. Within 6 h of intrauterine LPS treatment of pregnant mice, PSR-stained cross-linked collagen is reduced in the cervix while macrophages and neutrophils are more abundant compared to controls [35]. However, the effects of LPS on systemic progesterone are not clear during the initial 12 h after treatment [132]. Although a direct action by LPS on cell types in the cervix is not known, treatment to deplete pregnant mice of macrophages or to block inflammatory pathways forestalls preterm birth [133]. Whether such treatments decrease residency or alter the phenotype of macrophages in the prepartum cervix remains to be determined.

An essential question is whether cervix remodeling is the same for preterm birth as at term. Convergence of a variety of contributing stimuli could prematurely and differentially activate macrophages that reside in the cervix and promote remodeling through a final common mechanism (Figure 2C). By example, proinflammatory stimuli associated with infection [78] or activation of the complement pathway [134] advance cervix remodeling and induce preterm delivery in mice. However, the conclusion that LPS- and RU486-induced preterm cervix ripening through different mechanisms is premature because the cervix was collected from mice at different times after treatment (6 h versus 12 h) and treated on different days of pregnancy (d14 versus 15) though the same endpoint was compared between both treatment groups (gestation day 15.5 and midday gestation day 15) [135]. Current advances in understanding endpoints that characterize the remodeling process at terms can now be applied to assess the shift from phase 1 softening to phase 2 ripening following treatments that induce preterm cervix remodeling and advance birth.

## Perspective

Further evidence is needed to support the possibility that a final common mechanism for cervix remodeling at term is advanced with preterm birth. The cascade described in the schema in Figure 2 for the transition from a soft to ripe cervix may be defined by a threshold of proinflammatory molecules, crosstalk among resident cell types, and the counterbalance of opposing processes before term, e.g., wound healing, anti-inflammatory, oxidative stress countermeasures, and deposition of cross-linked collagen. With current understanding, it is premature to conclude that any one factor, including innervation, stretch, hypoxia, or proinflammatory mediators like LPS or PR inhibition, may regulate cervix remodeling.

Whether various factors to remodeling act to differentiate or recruit precursors to macrophages or directly stimulate resident macrophages remains to be determined. Certainly paracrine signals from PR receptive cells in the stroma may guide activities of macrophage phenotypes in the cervix. Understanding the integration of inputs in the final common pathway ripening may be critically dependent on signals from nonimmune cells to recruit, proliferate, differentiate, or activate macrophages. Molecules at each step of this cascade hold promise to diagnose the advance or delay in the remodeling process (Table 1). For example, the progress of remodeling may come from the finding that gene expression is upregulated for specific inflammatory molecules in the cervix of women and rodents [77,136]. This focus may also lead to novel therapeutic approaches to arrest ripening in women at risk for preterm labor or promote remodeling when complications of labor cause failure to progress to delivery at term.

**Table 1.** Key points to translate into clinical approaches to prevent early remodeling of the cervix in preterm birth

• Morphological characteristics associated with remodeling are temporally distinct and occur well before preterm changes in the uterus.
• Reduced systemic progesterone concentrations do not drive the transition from a soft to ripe cervix, but local changes might forecast structural degradation.
• PGF2α receptors and the genomic PR-A isoform are essential parts of the final common mechanism for remodeling.
• Prevalence and phenotype of macrophages in cervix may be sentinel for inflammatory process in remodeling.
• Strategies to block loss of progesterone efficacy, structural remodeling, and macrophage activation hold potential for diagnostic assessment of the progress of remodeling and for therapies to forestall preterm birth.

From a clinical perspective, a focus on cervix remodeling could serve as an early indicator of increased risk for preterm birth. Endpoints that include cell density, extracellular matrix collagen concentration, or structure from rodent models, along with identification of specific macrophage phenotypes or activities in the cervix, may prove useful to assess remodeling with the progress of pregnancy. For practitioners, the arrest of labor to prevent preterm birth is not a sufficient objective when the cervix has already ripened [137] because in the absence of a barrier to the vaginal microbiome, the fetus and mother are at risk for infection. Advances in technology may eventually correlate structure, morphology, cellular, or molecular parameters of the remodeling process with cervix length in women at risk for preterm labor. Novel treatments to block proinflammatory stimuli associated with critical macrophage activities [63] or other promoters may provide a useful intervention at critical breakpoints in the final common pathway for remodeling. A less promising approach to prevent preterm birth may be antibiotic treatments [138] since immune responses are predominantly associated with the adaptive rather than innate immune system. Ultimately, prevention of preterm birth is subordinate to the goal of improved perinatal and maternal morbidity and mortality, the benefits of which are to maximize health and well-being at the start of a new life.
